# The Management Dilemma of Leiomyosarcoma of the Ovary

**DOI:** 10.4021/wjon362w

**Published:** 2011-10-28

**Authors:** Elizabeth Jayne Goodall, Thumuluru Madhuri, Simon Butler Manuel

**Affiliations:** a Department of Gynae-Oncology, Royal Surrey County Hospital, Guildford, UK

**Keywords:** Leiomyosarcoma, Ovarian Neoplasm, Ovarian Sarcoma

## Abstract

Ovarian leiomyosarcomas (OLS) have a poor prognosis due to their late presentation and aggressive nature. This case illustrates that surgery alone can offer disease free survival in cases of early stage disease.

## Introduction

Pelvic sarcomas are renowned for being highly aggressive tumours with an inherently poor prognosis. Optimal surgery is a key element in the treatment of these tumours. We present a case of a woman with an ovarian leiomyosarcoma (OLS), now disease free 22 months after treatment.

## Case Summary

A 60 years old woman initially presented with left loin pain, loss of appetite and sterile pyuria. Past medical history included renal calculi and Crohn’s disease. Family history was positive for ovarian malignancy in her mother. An abdominal mass was found on clinical examination. Initial investigations revealed Ca125 at 20 (normal range: < 35 U/mL) and a CT abdomen and pelvis demonstrated a left complex solid ovarian mass causing bilateral hydronephrosis.

At laparotomy, frozen section of the mass suggested a pleomorphic spindle cell tumour. There was no ascites or evidence of peritoneal disease. A modified radical hysterectomy, bilateral salpingo-oophorectomy, omental biopsy and left pelvic lymph node sampling was performed and washings collected.

**Figure 1:**
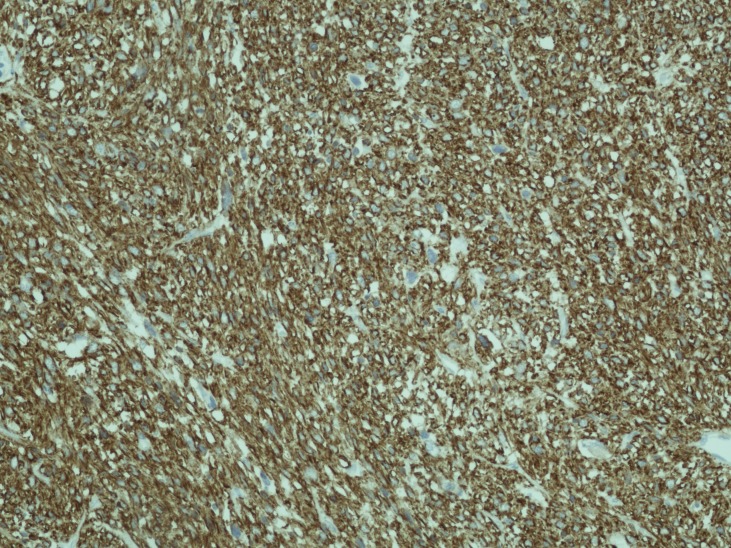
The specimen was strongly and diffusely positive to the markers H-Caldesmon, Desmin and smooth muscle Actin, focally and weakly positive to CD34 and negative to cytokeratins

The left ovary measured 140 x 70 x 75 mm with a smooth, fleshy white texture showing areas of haemorrhage and necrosis. Microscopically, there was complete replacement with malignant mesenchymal tumour, marked nuclear pleomorphism and multinucleated giant cells. The specimen was strongly and diffusely positive to the markers H-Caldesmon, Desmin and smooth muscle Actin, focally and weakly positive to CD34 and negative to cytokeratins (Fig. 1). The mitotic rate was over 10 per 10 high powered field (hpf). It was concluded to be a moderately differentiated leiomyosarcoma, confined within the ovarian capsule. The omentum, uterus, lymph nodes and washings sampled were tumour free.

The case was discussed at the supra-regional sarcoma meeting. In view of intact tumour resection with no metastatic disease, adjuvant therapy was not recommended but followed with close clinical monitoring and imaging. 22 months later, the patient remains disease free.

## Discussion

Less than 3% of all ovarian malignancies are primary sarcomas, the most common being rhabdomyosarcoma, fibrosarcoma, stromal cell sarcoma, with the rare leiomyosarcomas accounting for less than 0.1% [1]. The incidence of ovarian sarcoma to carcinoma is reported at 1:40 [2]. OLS primarily affects post-menopausal females, but have been reported even in adolescents [1, 3]. Typically, OLS are unilateral, large and frequently have distant metastases at presentation. This patient had a large unilateral tumour causing bilateral hydronephrosis but no distant metastases.

Presence of significant atypia with 5 or more mitoses per 10 hpf is suggestive of malignancy [4]. The pathogenesis of OLS is unclear but theories include malignant degeneration of smooth muscle present in the ovary (blood vessels and ligament attachments) or the migration and malignant transformation of uterine leiomyoma to the ovary [4].

Establishing standard treatment has proved difficult due to rarity of these cases and late presentation. In the majority of cases the disease confers a poor prognosis, with death ensuing within a year of diagnosis [2]. Monk et al recommend surgery as the mainstay of treatment for OLS, since adjuvant therapies are of unproven benefit especially in disease confined to the ovaries, as with our patient. Dixit et al suggest post-operative radiotherapy and chemotherapy in large tumour volumes to control local disease and avoid metastatic recurrence [2]. Rasmussen et al describe the role of repeated cyto-reductive surgery, hormonal treatment and adjuvant ifosfamide in their cases, attributing prolonged survival to surgical interventions [3].

### Conclusion

OLS are very rare tumours, typically affecting post-menopausal women and presenting at an advanced stage. Surgery remains the mainstay of treatment, as there is yet unproven benefit with adjuvant chemo- and radio-therapy. Although the overall prognosis is poor, early stage disease treated with optimal surgery may offer significant improvements in survival.
